# An Audit of Physical Healthcare in Mental Health Inpatients on Admissions Ward (Cedar Ward) in Llandough Hospital in Line With NCEPOD Guidance

**DOI:** 10.1192/bjo.2025.10648

**Published:** 2025-06-20

**Authors:** Olaide Oladosu, Rakesh Puli

**Affiliations:** Cardiff and Vale University Health Board, Cardiff, United Kingdom

## Abstract

**Aims:** Patients being admitted on mental health wards all have different forms of co-morbid physical health disorders needing complex care. They may require prompt transfer to medical wards for acute conditions and may need long-term monitoring for chronic ailments. National Confidential Enquiry into Patient Outcome and Death (NCEPOD) did a survey in 2022 focusing on the quality of physical health care delivered in psychiatry inpatients.

Aims were: To ascertain the percentage of patients that get a complete basic physical health examination.

To understand what physical health examinations are being undertaken during admission.

To check the proportion of patients that have their physical health conditions (co-morbidities) documented in their initial clerking.

Creating awareness on the gaps and potential improvements for physical healthcare on mental health wards.

**Methods:** Retrospective study.

Adult inpatients with mental health conditions admitted on Cedar Ward.

Duration of one-month period (28/10/2023–28/11/2023).

To compare the data with the NCEPOD report of 2022 (guidance).

Target of ≥ 40 patients.

**Results:** It was difficult to collect the data from records due to lack of uniformity in the documentation of physical health findings.

Physical health plan was made in 100% of patients, but only 72% got bloods done and 79% had a physical examination.

Despite DSU/MSU being planned by nursing staff for most of the patients only 15% got urine dip done.

70% got ECG done, but it was difficult to get this record as this was documented on different tabs on PARIS (Electronic patient records).

Among the different systems examined, surprisingly only 43% of the patients had a nervous system examination. Note that some patients had “moving all four limbs” as the only sign examined but this was not considered.

Of all healthcare providers, SHOs were the initial point of contact for assessment of physical health needs.

17% of patients did not have physical health conditions updated on electronic patient records platform (PARIS).

**Conclusion:** Firstly, there is a scope to improve the quality of physical health assessment in patients that get admitted on the wards.

Secondly a standardised structure for documentation can be helpful both for ease of access to information and to ensure that all our patients get a proper assessment of physical health needs.

Creating a standard proforma for physical health assessments in line with the guidance will act both as a guide and aid in uniformity in recording the findings.

